# Cerebral Vasospasm After Traumatic Subarachnoid Hemorrhage: A Narrative Review

**DOI:** 10.3390/medicina62081437

**Published:** 2026-07-24

**Authors:** Urška Hržič, Andreja Möller Petrun

**Affiliations:** 1 Department of Anesthesiology, Intensive Care and Pain Management, University Medical Centre Maribor, 2000 Maribor, Slovenia; urska.hrzic@gmail.com; 2 Faculty of Medicine, University of Maribor, 2000 Maribor, Slovenia

**Keywords:** subarachnoid hemorrhage, traumatic, vasospasm, intracranial, transcranial Doppler, treatment

## Abstract

Traumatic subarachnoid hemorrhage (tSAH) is a common complication of traumatic brain injury (TBI) and represents an important predictor of poor functional outcome. One of the most serious secondary complications is cerebral vasospasm. The pathophysiology of vasospasm is complex, involving the effects of blood breakdown products, inflammatory mediators, and direct mechanical injury to the cerebral vessels. Compared to aneurysmal subarachnoid hemorrhage (aSAH), vasospasm in tSAH typically occurs earlier, lasts for a shorter duration, and presents mostly with a milder clinical course. Due to its atypical clinical presentation and the presence of concurrent injuries, vasospasm is often not recognized in time and may manifest as neurological deterioration or new ischemic lesions on CT imaging. Timely recognition and appropriate management can significantly improve neurological outcomes. This paper presents key characteristics and differences between tSAH and aSAH, available diagnostic approaches, and treatment options, including nimodipine, milrinone, and stellate ganglion block. Owing to the lack of specific clinical guidelines for tSAH, current management strategies often rely on recommendations and experiences from aSAH. Further research is needed to better define risk factors for vasospasm following tSAH, optimize diagnostic pathways, and evaluate targeted treatment strategies.

## 1. Introduction

TBI remains one of the leading causes of morbidity, mortality, and long-term disability worldwide, particularly among younger adults and individuals of working age. Epidemiological data show that tSAH occurs in approximately 39–65% of patients with moderate to severe TBI and is associated with higher rates of intracranial hypertension, cerebral ischemia, and unfavorable neurological outcome [1,2]. Reported incidence varies substantially across studies, reflecting differences in diagnostic modalities and the increasing use of transcranial Doppler ultrasonography (TCD) for vasospasm screening [3].

One of the most significant secondary complications associated with tSAH is cerebral vasospasm, defined as a delayed and potentially reversible narrowing of large cerebral arteries that can impair cerebral perfusion and contribute to secondary brain injury [3,4,5]. While vasospasm has been extensively investigated in aSAH, where well-established pathophysiological mechanisms and clinical guidelines exist, the clinical relevance and optimal management of vasospasm in tSAH remain less clearly defined [3,4]. Importantly, vasospasm in tSAH appears to follow a different temporal pattern, may involve additional mechanisms such as mechanical vascular injury, and is often more difficult to identify due to sedation, impaired consciousness, or concomitant traumatic injuries [6,7,8,9].

Given the lack of standardized diagnostic criteria and targeted therapeutic recommendations, clinical management of vasospasm following tSAH is largely extrapolated from aSAH practice, despite distinct differences in underlying mechanisms and clinical behavior [3,4]. This discrepancy contributes to variability in clinical decision-making and may affect patient outcomes [3]. A clearer understanding of the pathophysiology, diagnostic challenges, and therapeutic considerations in tSAH is therefore essential to improve recognition and optimize patient outcomes.

## 2. Methodology

This narrative review was based on a targeted search of the available literature using PubMed and Google Scholar, followed by citation tracking of the reference lists of relevant publications to identify additional studies. The search included combinations of the following keywords: traumatic subarachnoid hemorrhage, aneurysmal subarachnoid hemorrhage, cerebral vasospasm, traumatic brain injury, transcranial Doppler, delayed cerebral ischemia, nimodipine, milrinone, stellate ganglion block, and neuromonitoring. Eligible publications included original studies, systematic reviews, meta-analyses, clinical guidelines, and other relevant publications. The review covers literature published between 1989 and 2025. Preference was given to recent publications. However, older landmark studies were included when considered essential for understanding the pathophysiology, diagnosis, or management of cerebral vasospasm following tSAH. Due to the limited availability of studies specifically addressing vasospasm after tSAH, evidence from aneurysmal subarachnoid hemorrhage was also included where considered clinically relevant, while acknowledging the limitations of extrapolating these findings to patients with tSAH. The final selection of references was based on their overall scientific relevance, methodological quality, and contribution to the objectives of this narrative review.

## 3. Pathophysiology

The pathophysiology of cerebral vasospasm after tSAH is not yet fully understood [4]. Due to the limited availability of studies specifically investigating tSAH, much of the current understanding is derived from evidence obtained in aSAH [10,11,12]. Although several mechanisms are likely shared between these two conditions, important differences may exist because they have different underlying causes [3,4]. Therefore, unless otherwise stated, the following discussion is primarily based on evidence from aSAH and applied to tSAH where appropriate.

In aSAH, blood breakdown products such as oxyhemoglobin, reactive oxygen species, and endothelin-1 play a central role in inducing vasoconstriction [13,14,15,16]. In tSAH, additional mechanisms are involved. Mechanical injury to the vessel wall or blast-related vascular stretch can independently trigger vasospasm, explaining why it may occur even in the absence of overt subarachnoid blood [3,4,17].

Recent evidence indicates that cerebral vasospasm after aSAH is not merely the result of smooth muscle contraction but rather a complex cascade involving endothelial dysfunction, oxidative stress, microthrombosis, inflammation, and impaired nitric oxide (NO) signaling [12]. The breakdown of erythrocytes in the subarachnoid space releases oxyhemoglobin, which scavenges NO and promotes vasoconstriction through activation of Rho-kinase and protein kinase C pathways. Inflammatory mediators such as IL-6, IL-1β, and TNF-α further contribute to blood–brain barrier disruption, cortical spreading depolarization, and delayed cerebral ischemia (DCI) [10,18,19,20,21,22,23,24]. Cortical spreading depolarizations are increasingly recognized as an important contributor to DCI. They cause cerebral ischemia by increasing metabolic demand in injured tissue unable to compensate with increased perfusion [10]. In addition, impaired clearance of hemoglobin degradation products due to dysfunction of the glymphatic and meningeal lymphatic systems exacerbates secondary brain injury [10,11,25].

The combined effects of vasospasm, endothelial dysfunction, neuroinflammation, microthrombosis, impaired microcirculation, cortical spreading depolarizations, and impaired autoregulation may ultimately result in DCI. DCI represents one of the most important secondary complications following aSAH and is a major determinant of neurological outcome [10]. It is defined by the American Heart Association as the occurrence of a new focal neurological deficit or a decrease of at least two points on the Glasgow Coma Scale (GCS) lasting for at least one hour, which cannot be explained by other causes. Imaging-defined DCI refers to the development of new cerebral infarction not attributable to other factors. Although DCI has been extensively investigated in aSAH, accumulating evidence suggests that it also occurs after tSAH and is associated with worse functional outcome and increased mortality [26].

The relative contribution of these mechanisms to DCI after tSAH remains uncertain because of the limited number of dedicated studies. Nevertheless, current evidence suggests that similar mechanisms are likely involved in secondary brain injury after tSAH, although their individual roles and interactions remain to be established in dedicated clinical studies [26].

## 4. Comparison of Vasospasm in tSAH and aSAH

Cerebral vasospasm shows distinct temporal and clinical characteristics depending on whether it follows traumatic or aneurysmal subarachnoid hemorrhage. In aSAH, vasospasm most frequently develops between days 4 and 14, rarely before day 3 [6,9,17]. In contrast, vasospasm after tSAH often emerges much earlier, frequently within the first 48 h after injury. Its duration is generally shorter—most cases resolve within two weeks, and in some instances within only a few days—while in aSAH vasospasm may persist for three weeks or longer [8,17,27,28,29].

The pattern of blood distribution within the subarachnoid space appears to play a pivotal role in the development and clinical course of vasospasm [4]. In tSAH, bleeding is often diffuse across the convexities, while in aSAH it is typically concentrated in the basal cisterns. These anatomical differences affect cerebrospinal fluid circulation and the clearance of blood breakdown products, which in turn influence the risk and severity of vasospasm. Faster resorption of blood in tSAH may explain the usually milder clinical course, whereas aSAH more frequently results in territorial ischemia visible on CT imaging [4,30].

The correlation between the amount of blood and the risk of vasospasm also differs [6]. In aSAH, a strong association exists between blood volume in the basal cisterns and vasospasm severity [7,31]. By contrast, in tSAH, this correlation is less consistent. For example, Gaetani et al. found that the Fisher scale did not reliably predict vasospasm in tSAH but was instead associated with long-term neurological outcome as measured by the Glasgow Outcome Scale (GOS) [32]. These findings indicate that vasospasm following TBI is influenced by additional mechanisms beyond the volume of subarachnoid blood. Consequently, the Fisher grade appears to have lower predictive value for vasospasm in tSAH than in aSAH and should be interpreted with caution in this population [4,17].

Moreover, vasospasm in TBI may develop even in the absence of radiologically proven tSAH, suggesting that factors such as mechanical vascular injury or low admission GCS may be stronger predictors than subarachnoid blood volume alone [8,17,33]. Clinical recognition of vasospasm in tSAH is further complicated by sedation, reduced consciousness, and concomitant injuries [4,8].

The main differences between vasospasm in aSAH and tSAH are summarized in Table 1, highlighting the timing, duration, mechanisms, distribution of blood, and diagnostic reliability of TCD .

## 5. Risk Stratification

Identifying patients at increased risk of post-traumatic vasospasm may facilitate targeted surveillance and earlier diagnosis. However, reliable risk stratification remains challenging because most available evidence originates from retrospective observational studies, often involving relatively small cohorts from single centers, with heterogeneous patient populations and variable diagnostic criteria [4].

One of the earliest studies investigating predictors of post-traumatic vasospasm was conducted by Zubkov et al., who prospectively evaluated 90 patients with TBI using daily TCD. The authors found that vasospasm occurred predominantly in patients with severe TBI (GCS 8 or less) and was strongly associated with severe tSAH on the initial CT scan. Patients with lower GCS scores at admission also demonstrated a significantly higher incidence of vasospasm (50% of patients with GCS 3–4). In addition, epidural and subdural hematomas were associated with an increased risk of vasospasm. Although this study provided important early insights, its findings should be interpreted cautiously because of the relatively small sample size and single-center design [17].

Subsequent studies have reported similar associations while identifying additional potential predictors. In their review, Al-Mufti et al. [3] summarized evidence suggesting that severe tSAH remains the most consistently reported imaging predictor of post-traumatic vasospasm [4,8,17]. Other reported risk factors included intracerebral hematoma, epidural and subdural hematomas, lower admission GCS scores, higher Injury Severity Score, fever, greater extent of cerebral injury, hemorrhagic contusions, and traumatic pseudoaneurysms. Nevertheless, these associations have not been consistently reproduced across studies, reflecting differences in study design, patient populations, diagnostic methods, and definitions of vasospasm. Consequently, no validated risk stratification model currently exists for patients with tSAH [4].

## 6. Diagnostic Approaches

### 6.1. Clinical Limitations and Need for Multimodal Monitoring

The clinical recognition of vasospasm in patients with TBI is often limited, as neurological deterioration may be masked by sedation, impaired consciousness, or concomitant injuries [4,8]. For this reason, timely diagnosis relies heavily on neuroimaging and neuromonitoring modalities [36].

### 6.2. Transcranial Doppler Ultrasonography

TCD is a bedside, non-invasive, repeatable, inexpensive, reliable method, and represents the primary diagnostic tool for detecting cerebral vasospasm [34]. It is most accurate in the middle cerebral artery (MCA), intracranial internal carotid artery (ICA), and basilar artery (BA), but less reliable in the anterior and posterior cerebral arteries. Because systolic velocities are strongly influenced by hemodynamic factors, the diagnosis of vasospasm is based on mean flow velocity (MFV). Regular monitoring, at least every other day in aSAH protocols, is recommended to ensure timely detection. Diagnostic accuracy is enhanced by the Lindegaard ratio (LR), which compares MCA MFV with extracranial ICA flow. An MCA vasospasm is unlikely when MFV < 120 cm/s and LR < 3, while severe vasospasm is highly likely with MFV > 200 cm/s and LR > 6. Similarly, in the vertebrobasilar system, a BA/VA (vertebral artery) ratio > 2 is indicative of vasospasm. Combined sensitivity and specificity for MCA vasospasm detection with TCD and LR reach 90% and 100%, respectively [37].

### 6.3. Computed Tomography-Based Imaging

CT angiography (CTA) provides rapid and reliable visualization of proximal intracranial arteries [34,38]. Most available evidence regarding CTA diagnostic accuracy originates from aSAH cohorts and has not been extensively validated in patients with tSAH. Therefore, these diagnostic performance estimates should be interpreted with caution when extrapolated to the tSAH population. In patients with aSAH, CTA demonstrates high sensitivity (up to 91%) for detecting central cerebral vasospasm and an estimated specificity of approximately 95% when compared with digital subtraction angiography, although diagnostic performance is reduced in distal vascular territories [34,39,40]. CT perfusion further evaluates cerebral blood flow changes, helping distinguish symptomatic vasospasm from established infarction [41].

### 6.4. Advanced Neuromonitoring Techniques

Additional neuromonitoring techniques can support the early detection of cerebral ischemia and guide treatment decisions. These modalities primarily assess regional cerebral oxygenation and metabolism, providing physiologic insight that may precede radiologic or clinical deterioration. Their greatest value lies in their complementary use within a multimodal monitoring strategy [42].

#### 6.4.1. Electroencephalography

Continuous electroencephalography (EEG), particularly quantitative EEG (qEEG), can reveal ischemic changes—such as reduced relative alpha variability, decreased alpha/delta ratio, and diminished absolute alpha power—more than 24 h before clinical deterioration [43,44]. Although not specific for vasospasm alone, qEEG is a valuable adjunct in high-risk patients [45].

#### 6.4.2. Near-Infrared Spectroscopy

Near-infrared spectroscopy (NIRS) is a non-invasive method for monitoring regional cerebral oxygenation (rSO_2_), allowing continuous bedside measurement of cortical oxygen saturation. It is based on measuring oxygenated and deoxygenated hemoglobin in mixed blood, particularly in the frontal lobes [46]. In the study by Park et al., NIRS proved to be a useful method for early detection of cerebral ischemia resulting from vasospasm after aSAH. A decrease in rSO_2_ of 14.7% or more from baseline enabled the detection of vasospasm-related ischemia with high sensitivity and specificity. Despite limitations such as superficial measurement depth and the possibility of artifacts, NIRS represents a promising complementary tool for early recognition of vasospasm [35].

#### 6.4.3. Brain Tissue Oxygen Monitoring

Brain tissue oxygen monitoring (PbtO_2_) is an invasive neuromonitoring method in which a probe is inserted into brain tissue considered at risk of vasospasm. The measurement allows continuous monitoring of brain tissue oxygenation in the immediate vicinity of the probe [47]. A decrease in PbtO_2_ values raises suspicion of vasospasm and indicates the need for further diagnostic evaluation. However, this method has important limitations, primarily that it measures oxygenation only within a limited region of the brain, which may lead to overlooking ischemic areas elsewhere. For this reason, it is not reliable as a standalone diagnostic tool [48].

#### 6.4.4. Cerebral Microdialysis

Cerebral microdialysis is an invasive neuromonitoring method that involves the insertion of a catheter with a semipermeable membrane into brain tissue, allowing continuous sampling of extracellular fluid [49,50]. This technique provides real-time information on cerebral metabolism, including levels of markers such as lactate, pyruvate, glucose, and glutamate. In the context of subarachnoid hemorrhage, changes in these metabolic parameters may reflect evolving cerebral ischemia or secondary injury processes [50,51,52]. However, the usefulness of microdialysis is limited by its focal nature, as it provides metabolic information only from the region surrounding the catheter tip. Therefore, while microdialysis may serve as a valuable adjunct within a multimodal monitoring strategy, it is insufficient as a standalone diagnostic tool for detecting vasospasm [50].

## 7. Prevention and Treatment of Vasospasm

Because evidence specifically addressing cerebral vasospasm after tSAH remains limited [4], the following section includes both studies performed directly in patients with tSAH and evidence from aSAH.

In patients with aSAH, well-established clinical guidelines emphasize early prevention of vasospasm [34]. The cornerstone is prophylactic therapy with the calcium channel blocker nimodipine, administered for 21 days, which has been shown to reduce the incidence of infarction and improve outcomes [3,34,53]. It can be given orally or intravenously, though oral administration is generally recommended unless contraindicated [53]. Nimodipine acts by blocking dihydropyridine calcium channels in vascular smooth muscle, thereby reducing contractility and limiting vasospasm [54,55,56,57].

The treatment of vasospasm in aSAH has shifted from the historical “triple-H therapy” (hypervolemia, hypertension, hemodilution) to targeted induced hypertension with normovolemia, sometimes combined with intra-arterial vasodilators or balloon angioplasty. Among pharmacological options, milrinone, a phosphodiesterase III inhibitor, has both vasodilatory and inotropic effects. It can be administered intravenously or intra-arterially during digital subtraction angiography (DSA) in severe cases. Dose escalation is limited by side effects such as hypotension and tachycardia (8). Stellate ganglion block (SGB) has also been explored as a rescue therapy in refractory vasospasm, with studies in aSAH showing improved cerebral hemodynamics and neurological outcomes [58,59].

In contrast, no specific guidelines exist for tSAH. Clinical management often extrapolates from aSAH despite important pathophysiological and clinical differences. Triple-H therapy may be harmful in TBI patients due to increased risk of cerebral edema or rebleeding. Similarly, calcium channel blockers may lower blood pressure, further compromising cerebral perfusion [3,60].

Evidence on nimodipine in tSAH is mixed [61]. Some randomized trials demonstrated reduced radiological vasospasm and fewer unfavorable outcomes at six months, while others found no significant difference compared to placebo [62,63]. Trials with nicardipine reported improved cerebral blood flow but no consistent clinical benefit [64].

Early randomized trials evaluating nimodipine in patients with tSAH suggested a potential benefit. Head Injury Trial (HIT) 3 reported fewer poor outcomes (defined as death, vegetative state, or severe disability on the GOS at 6 months) in patients treated with nimodipine. However, the subsequent and substantially larger HIT 4 trial (577 patients) failed to confirm this benefit. In the systematic review by Vergouwen et al., which included data from four randomized trials and 1074 patients with tSAH, poor neurological outcome occurred in 39% of patients treated with nimodipine and 40% of placebo-treated patients (OR 0.88, 95% CI 0.51–1.54), while mortality was also similar between groups (26% vs. 27%; OR 0.95, 95% CI 0.71–1.26). The authors concluded that the available evidence does not support a significant benefit of routine nimodipine therapy in patients with tSAH. The conflicting findings across trials likely reflect substantial differences in sample size, methodological quality, and patient selection, with much of the evidence originating from subgroup analyses of head injury trials rather than studies specifically designed for patients with tSAH. Therefore, adequately powered multicenter randomized trials specifically enrolling patients with tSAH are still needed before routine nimodipine therapy can be recommended [61].

A randomized clinical trial by Fathi and Medhat suggests a potential role for combined therapy. They compared oral nimodipine plus triple-H therapy with oral nimodipine plus intravenous milrinone in patients with confirmed vasospasm after tSAH. The combination of nimodipine and milrinone was associated with higher brain oxygenation, improved Glasgow Coma Scale scores at 14 days, shorter ICU and hospital stay, and better long-term outcomes. Although hypotension and electrolyte disturbances were more common, serious complications such as cerebral infarction were less frequent than with triple-H therapy [65].

Despite these promising findings, the available evidence supporting intravenous milrinone for vasospasm after tSAH remains limited. This randomized clinical trial included just 30 patients and was conducted at a single tertiary care center, limiting statistical power and generalizability of the findings. Furthermore, only patients with mild-to-moderate tSAH (World Federation of Neurological Surgeons grades I–III and Fisher grades II–III) were included, while patients with more severe tSAH, significant comorbidities, or hemodynamic instability were excluded. Consequently, the results may not be generalized to the broader population of patients with tSAH. In addition, the comparing group received triple-H therapy, a strategy that is no longer routinely recommended in neurocritical care because of limited evidence of benefit and potential harmful effects. Although the study demonstrated improved cerebral oxygenation, neurological recovery, and functional outcomes with milrinone, these findings require confirmation in larger, multicenter randomized trials before routine clinical implementation can be recommended in patients with tSAH [3,65].

In recent years, SGB has gained attention as a promising therapeutic option. Although studied mainly in the context of aSAH, it may also be useful in tSAH. Its mechanism is based on sympathetic blockade, leading to vasodilation of cerebral arteries and improved perfusion [66]. Studies have shown that SGB can rapidly improve cerebral blood flow and neurological status, including GCS, with effects lasting up to 12–24 h. The procedure is usually performed at the C6–C7 level under ultrasound guidance using local anesthetics such as bupivacaine or ropivacaine. While generally safe, potential complications include hoarseness, dysphagia, hematoma, phrenic nerve block, or accidental epidural injection, and Horner’s syndrome, which typically confirms successful block [58,59,66].

Clinical studies in aSAH have demonstrated improvements in cerebral blood flow velocity, angiographic vessel diameter, cerebral perfusion, and neurological status following ultrasound-guided SGB. The available evidence is based on small single-center prospective studies involving only 15 and 20 patients, respectively, without randomized controlled comparisons, making the findings susceptible to selection bias and limiting statistical power and external validity. Furthermore, those clinical studies have exclusively enrolled patients with aSAH, whose pathophysiology, natural history, and response to treatment differ from those of tSAH. Consequently, the efficacy and safety of SGB in patients with tSAH remain uncertain, and current evidence is insufficient to support its routine use. Prospective, adequately powered, multicenter randomized controlled trials specifically enrolling patients with tSAH are required before SGB can be recommended as a standard treatment for vasospasm after tSAH [58,59].

Overall, the treatment of vasospasm after tSAH remains challenging because high-quality evidence is scarce. Consequently, further prospective multicenter studies are required to establish evidence-based treatment recommendations and standardized management protocols for patients with tSAH-associated vasospasm [3,4,36].

**Figure 1:**
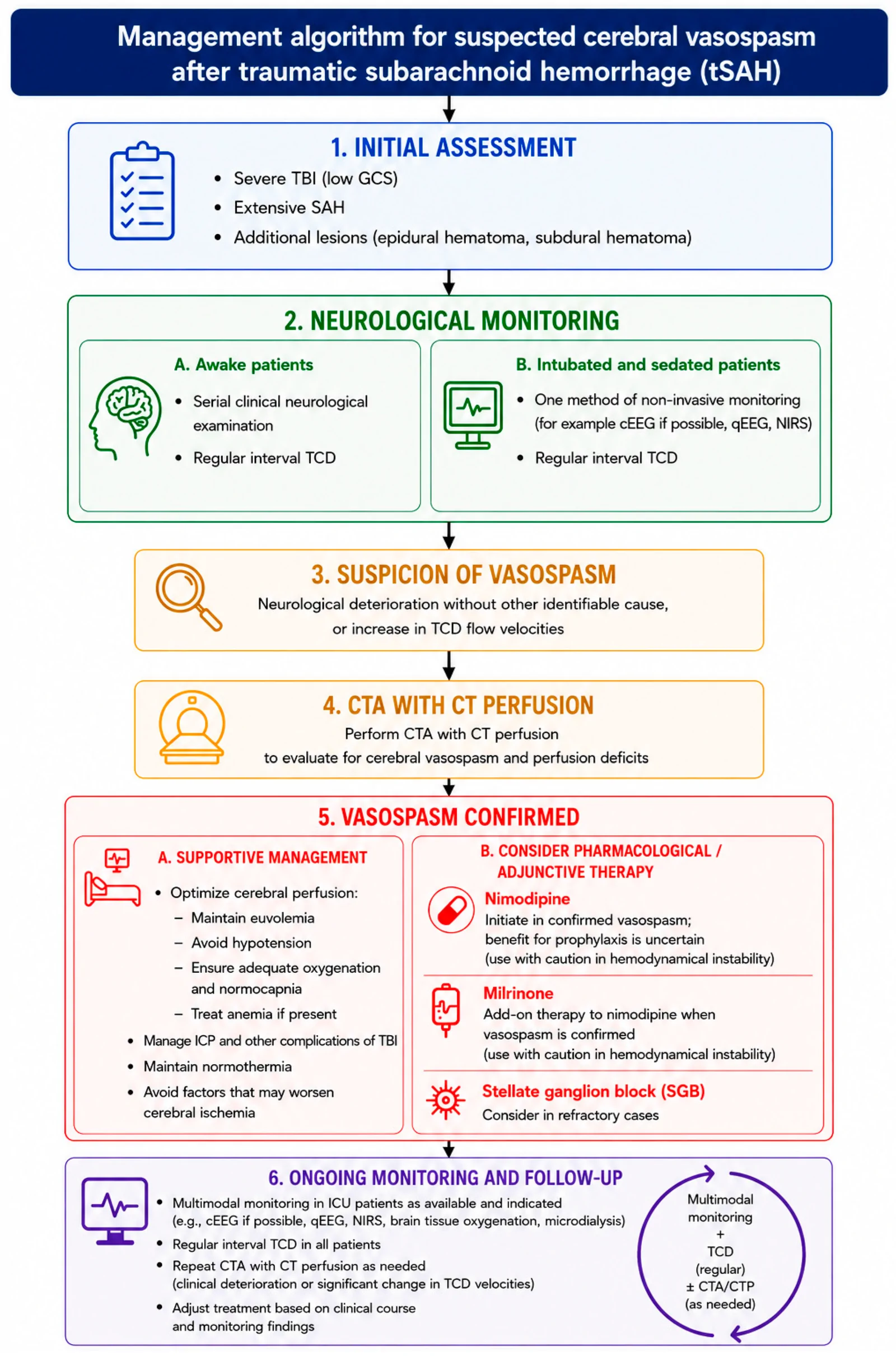
Proposed diagnostic and management algorithm for suspected cerebral vasospasm after tSAH. Legend: TBI, traumatic brain injury; tSAH, traumatic subarachnoid hemorrhage; GCS, Glasgow Coma Scale; cEEG, continuous electroencephalography; qEEG, quantitative electroencephalography; NIRS, near-infrared spectroscopy; TCD, transcranial Doppler ultrasonography; CTA, computed tomography angiography; CTP, computed tomography perfusion; ICP, intracranial pressure; SGB, stellate ganglion block; ICU, intensive care unit. Figure created with the assistance of ChatGPT Version 5.5 (OpenAI).

Given the limited evidence specific to tSAH and the absence of standardized management guidelines, Figure 1 summarizes a diagnostic and therapeutic approach based on the currently available evidence discussed in this review.

## 8. Knowledge Gaps and Future Directions

Despite growing recognition of cerebral vasospasm after tSAH, several important knowledge gaps remain [3,4]. The true incidence of clinically significant vasospasm, optimal screening strategies, and the identification of patients most likely to benefit from monitoring or intervention remain incompletely defined [4,17]. Furthermore, most therapeutic approaches are extrapolated from aSAH, while high-quality evidence specific to tSAH remains limited [3,4].

Future research should focus on prospective multicenter studies using standardized diagnostic criteria and clinically meaningful outcome measures. Priorities include validating risk stratification tools, defining the role and timing of multimodal neuromonitoring, and evaluating pharmacological therapies and other emerging treatment strategies in tSAH-specific populations. Ultimately, the development of evidence-based clinical guidelines will depend on adequately powered multicenter prospective studies addressing these remaining knowledge gaps [3,4,61].

## 9. Conclusions

Cerebral vasospasm remains an important but frequently underrecognized complication of tSAH. Although its incidence may be as high as 68% in radiological studies, the clinical impact is less predictable compared with aSAH [3,4]. In tSAH, vasospasm develops earlier, is usually shorter in duration, and often presents with a milder clinical course. Nevertheless, it can contribute substantially to secondary brain injury and adverse outcomes in selected patients [3,36].

The absence of standardized diagnostic criteria and therapeutic guidelines specifically tailored to tSAH patients continues to represent a major challenge. Current management strategies are often extrapolated from aSAH protocols, despite important differences in pathophysiology, timing, and clinical manifestation. This discrepancy contributes to variability in practice and highlights the need for more individualized approaches [3,4,12]. While nimodipine is well established as prophylaxis in aSAH, its benefit in tSAH is less consistent, and other approaches, including milrinone and stellate ganglion block, have produced mixed but promising results [58,59,65].

Until tSAH-specific evidence-based guidelines become available, a pragmatic approach may be to identify patients at higher risk of cerebral vasospasm based on clinical and radiological features, such as severe TBI, extensive tSAH, or progressive neurological deterioration. In these patients, serial neurological assessment combined with multimodal monitoring, including TCD and CT-based imaging when clinically indicated, may facilitate earlier recognition of vasospasm and secondary cerebral ischemia [3,4,6,17,36,37,38,39,40]. Non-invasive neuromonitoring techniques may be particularly useful in patients with persistent impaired consciousness or sedation, where clinical detection of neurological deterioration is limited and early identification of vasospasm may facilitate timely interventions [35,42,43,44,45,46,47,48,49,50,51,52].

Ultimately, developing standardized, evidence-based recommendations will be crucial to optimize care, reduce neurological morbidity, and improve long-term outcomes in patients with tSAH.

## Figures and Tables

**Table 1:** Comparison of vasospasm characteristics in aSAH and tSAH.

Parameter	aSAH	tSAH	Representative References
Earliest onset	Day 3 (range 4–14)	Day 1 (range 2–6)	[3,4,6,17]
Time of peak MFV	Days 5–14	Days 5–7 (sometimes earlier)	[3,4,9]
Incidence on TCD	38–45%	19–68%	[3,4,8,17,29]
Incidence on angiography	43.2%	18.6–41%	[3,4,6,7,17]
Symptomatic vasospasm (with radiological confirmation)	17–40%	3.9–16.6%	[3,4,6,7,17]
Location of SAH	Basal cisterns	Convexities, tentorium	[3,4,30]
Duration of vasospasm	Up to 3 weeks or longer	Often shorter, sometimes <5 days	[3,4,6,17]
Main mechanisms	Blood breakdown products	Blood + mechanical injury	[3,4,29,30]
Ischemic pattern	Territorial infarction	Often atypical, non-territorial	[3,4,30]
Diagnostic value of TCD	High (well validated)	Useful, less validated	[3,4,34,35]

aSAH, aneurysmal subarachnoid hemorrhage; tSAH, traumatic subarachnoid hemorrhage; MFV, mean flow velocity; TCD, transcranial Doppler ultrasonography.

## Data Availability

No new data were created or analyzed in this study. Data sharing is not applicable to this article.

## Abbreviations

The following abbreviations are used in this manuscript:

aSAH	Aneurysmal subarachnoid hemorrhage
BA	Basilar artery
cEEG	Continuous electroencephalography
CTA	Computed tomography angiography
CTP	Computed tomography perfusion
DCI	Delayed cerebral ischemia
DSA	Digital subtraction angiography
EEG	Electroencephalography
GCS	Glasgow Coma Scale
GOS	Glasgow Outcome Scale
HIT	Head Injury Trial
ICA	Internal carotid artery
ICP	Intracranial pressure
IL	Interleukin
LR	Lindegaard ratio
MCA	Middle cerebral artery
MFV	Mean flow velocity
NIRS	Near-infrared spectroscopy
NO	Nitric oxide
PbtO_2_	Brain tissue oxygen monitoring
qEEG	Quantitative electroencephalography
rSO_2_	Regional cerebral oxygen saturation
SAH	Subarachnoid hemorrhage
SGB	Stellate ganglion block
TBI	Traumatic brain injury
TCD	Transcranial Doppler ultrasonography
tSAH	Traumatic subarachnoid hemorrhage
TNF-α	Tumor necrosis factor alpha
VA	Vertebral artery
